# dbEM: A database of epigenetic modifiers curated from cancerous and normal genomes

**DOI:** 10.1038/srep19340

**Published:** 2016-01-18

**Authors:** Jagpreet Singh Nanda, Rahul Kumar, Gajendra P. S. Raghava

**Affiliations:** 1 Bioinformatics Centre, CSIR-Institute of Microbial Technology, Chandigarh-160036, India

## Abstract

We have developed a database called dbEM (**d**ata**b**ase of **E**pigenetic **M**odifiers) to maintain the genomic information of about 167 epigenetic modifiers/proteins, which are considered as potential cancer targets. In dbEM, modifiers are classified on functional basis and comprise of 48 histone methyl transferases, 33 chromatin remodelers and 31 histone demethylases. dbEM maintains the genomic information like mutations, copy number variation and gene expression in thousands of tumor samples, cancer cell lines and healthy samples. This information is obtained from public resources *viz*. COSMIC, CCLE and 1000-genome project. Gene essentiality data retrieved from COLT database further highlights the importance of various epigenetic proteins for cancer survival. We have also reported the sequence profiles, tertiary structures and post-translational modifications of these epigenetic proteins in cancer. It also contains information of 54 drug molecules against different epigenetic proteins. A wide range of tools have been integrated in dbEM e.g. Search, BLAST, Alignment and Profile based prediction. In our analysis, we found that epigenetic proteins DNMT3A, HDAC2, KDM6A, and TET2 are highly mutated in variety of cancers. We are confident that dbEM will be very useful in cancer research particularly in the field of epigenetic proteins based cancer therapeutics. This database is available for public at URL: http://crdd.osdd.net/raghava/dbem.

Epigenetics is the study of heritable changes in cellular phenotype without any change in the DNA sequence. It involves DNA methylation or posttranslational modification of histones by the activity of chromatin modifiers namely DNA methyl transferases (DNMTs), histone methyltransferases (HMTs), histone demethylases (HDMs), histone deacetylases (HDACs), histone acetyl transferases (HATs) and chromatin remodelers. Epigenetic alterations are dynamic in nature and even influenced by environment^1^,^2^. Epigenetic modifications play a major role in gene regulation processes such as transcription, DNA repair and replication. It has been shown in past that abnormal expression patterns or genomic alterations in chromatin regulators can lead to cancer. Coupling of next generation sequencing (NGS) techniques to chromatin immunoprecipitation (ChIP-Seq) for analysis of cancer genomes has provided a list of somatic mutations in epigenetic regulators and altered DNA methylation patterns at CpG islands patterns that drive oncogenesis^3^,^4^,^5^,^6^.

Genetic mutations and altered gene expression of chromatin modifiers in vast array of cancers not only implies their causative role but also highlight novel potential targets for cancer therapy. Recently, Food and Drug Administration (FDA) approved the use of vorinostat and romidepsin (HDAC inhibitors); azacitidine and decitabine (DNMT inhibitors) for the treatment of cutaneous T cell lymphoma and myelodysplastic syndrome^7^,^8^,^9^. As epigenetic proteins are druggable^10^,^11^, there is a need of resources that allow comprehensive analysis of the epigenetic landscape in cancer to identify potential targets. To the best of our knowledge, there is no existing database, which compiles the information pertinent to the mutation/variations, expression levels, and gene essentiality data of epigenetic proteins (chromatin regulators) in cancer studies.

In the present study, we developed a database called dbEM, which maintains the extensive detailed information of each epigenetic modifier. This is a curated database where genomic information is collected from the literature and other existing databases. We hope that this database will be an informative tool for the researchers working in this field.

## Objective of the Database

**dbEM** is dedicated to study the role of epigenetic proteins in oncogenesis. In order to analyze the alterations and imbalances in epigenomic landscape in cancer, genomic information pertaining to mutational status, copy number variation and expression level of epigenetic proteins in tumor samples and cell lines have been compiled. Gene essentiality data and expression levels of epigenetic proteins in various cancers are also included in this database. dbEM offers a user-friendly web interface, which also gives basic information such as tertiary structure, sequence alignment and profiles, post-translational modifications. Apart from this, it also provides brief information about the use of small inhibitors (either alone or in combination) targeting various epigenetic proteins. This database will help researchers to identify the altered epigenetic proteins that may have a role in oncogenesis and could be explored as therapeutic targets.

## Systems and Methods

### Database architecture and web interface

**Figure 1:**
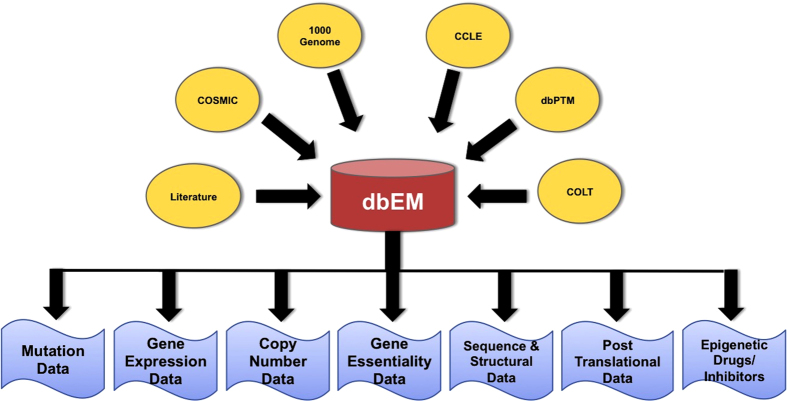
Architecture of dbEM database.

Apache HTTP Server and MySQL server were used to build dbEM. MySQL server works at the backend that helps to retrieve and store data. Front-end web interface was developed by integrated use of PHP, HTML, CSS and Java. Scripts for web database interface were written using PERL programming language. dbEM comprises various types of information about each epigenetic protein collected from different resources as shown in Fig. 1.

## Data Acquisition

### Epigenetic Proteins

The criterion for selection of epigenetic proteins was their involvement as major players of epigenetic mechanisms in gene regulation and cancer on the basis of earlier published literature. On the basis of above criteria, we selected 167 epigenetic proteins and compiled available data for these proteins to develop dbEM. These proteins were broadly classified as DNA methyl transferases (DNMTs), histone deacetylases (HDACs), histone acetyltransferases (HATs), histone methyltransferases (HMTs), histone demethylases (HDMs) and chromatin remodelers.

### Genomic Data

**Figure 2:**
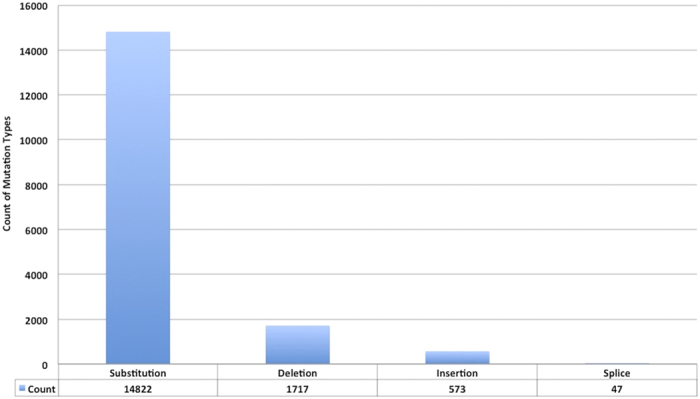
Bar graph showing the counts of various types of mutations in epigenetic proteins.

**Figure 3:**
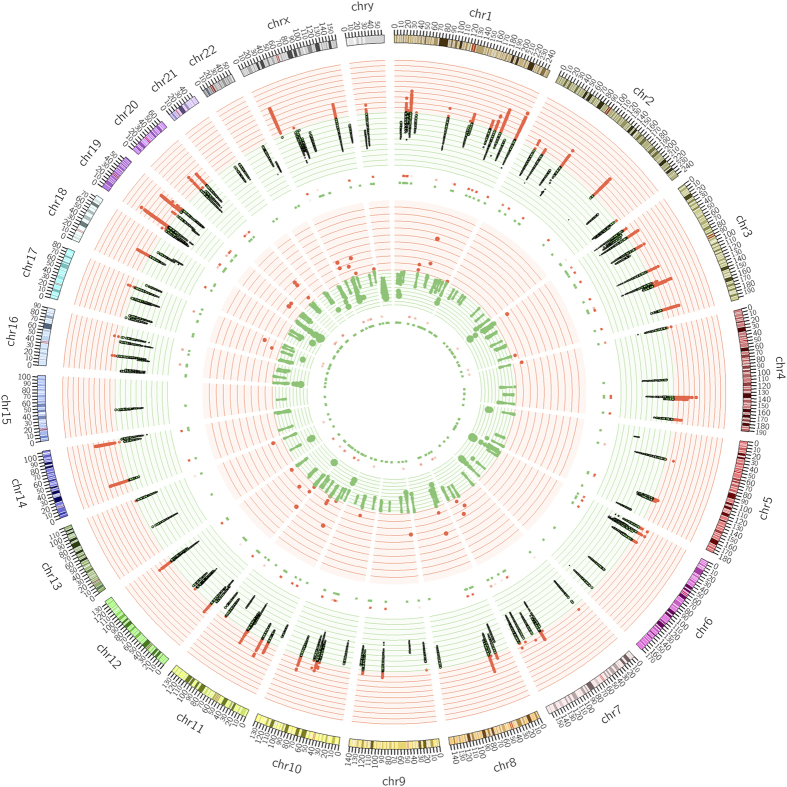
Circos plot depicting the gene expression and copy number variation. Moving from outer to inner circles, first circle represents the chromosomes on which epigenetic genes have been mapped. Gene expression data has been represented in second and third circles as scatter diagram and heat map, respectively (red and green dots denote gene expression values greater and less than 10, respectively). Fourth and fifth circles represent copy number variation (CNV) as scatter diagram and heat map, respectively (red and green dots have values greater and less than 2, respectively). Each dot represents a cancer cell line.

Cancer Cell Line Encyclopedia (CCLE)^12^ and Catalogue of Somatic Mutation in Cancer (COSMIC)^4^ are the two primary resources used to retrieve the mutation data of these proteins. We have filtered out a total of 17159 mutations present in 167 epigenetic proteins from CCLE and COSMIC data. Out of all kinds of mutations, substitution was the major type of mutation as shown in Fig. 2. For expression and copy number variation (CNV) data, CCLE expression data based on Affymetrix U133 plus array (normalized by RMA technique using quantile normalization) and log2 CNV data from Affymetrix SNP 6.0 arrays was utilized. Circos plot^13^ highlights the gene expression and CNV data in Fig. 3.

### Gene Essentiality Data

In order to investigate the essentiality of particular genes encoding for above-mentioned epigenetic proteins for survival of cancer, we compiled shRNA dropout profiles from COLT-cancer database^14^. This database contains gene essentiality data of around 16000 genes in 72 cancer cell lines of ovarian and pancreatic tissues. Essentiality of a particular gene is reflected in terms of GARP score and significant P-value. GARP score is a measure of shRNA dropout rate and less GARP score (more negative) represents more essentiality.

### Tertiary Structure and Structural Domains

In order to assist drug designing of small molecules targeting epigenetic proteins, structural information about these proteins is also compiled in dbEM. In spite of the availability of PDB structures of around 109 proteins, we used HH suite 2.0.16^15^ and Modeller 9.13^16^ to model the structure of all 167 epigenetic proteins. Modeled structures can be visualized in Jmol applet and PDB files can be downloaded. We have provided links to all available structures in the PDB. We have also mapped the Pfam^17^ and Superfamily^18^ domains to identify characteristic domains of epigenetic proteins. FASTA sequences of proteins were used as a query to map the Pfam and Superfamily domains with default settings. Finally, we mapped 837 Pfam domains and 371 Superfamily domains in 167 epigenetic proteins.

### Sequence Alignment

dbEM allows user to align the sequence of epigenetic proteins in different ways. Information about normal variations of epigenetic proteins was taken from 1000 genome project in VCF format. Variations of each protein were extracted from VCF file and mapped onto their wild-type sequences to create different variants. Finally, these variants were aligned with wild-type proteins. In addition, wild-type sequences were also aligned with mutants available in CCLE and COSMIC. To get evolutionary information about these epigenetic proteins, their homologous sequences in different species (obtained from NCBI) were aligned and an evolutionary tree was generated using ClustalW^19^ and Jalview^20^ were used for sequence alignment and better visualization of data respectively.

### HMM and PSSM Profiles

HMM and PSSM profiles were generated for each protein which give a conservation score at each position of protein. ‘Jackhmmer’ and ‘hmmbuild’ modules of HMMER^21^ software were used to generate HMM profiles whereas PSSM profiles were made by using ‘blastpgp’ and ‘makemat’ modules of BLAST software^22^. Three databases were used to create these profiles *viz*. UniProt database, mutated sequences database and normal variant database.

### Post-translational Modifications

Since earlier studies have shown that functioning of various proteins is regulated by different post-translational modifications such as phosphorylation, acetylation, ubiquitylation, sumoylation^23^,^24^,^25^. PTM information was also included in this database. dbEM provides complete information about position, amino acid (which is modified) and type of modifications for all the epigenetic proteins compiled from dbPTM^26^.

### Epigenetic drugs and Inhibitors

Recently, FDA approved the use of epigenetic drugs such as vorinostat and azacitidine for treatment of cutaneous T cell lymphoma and myelodysplastic syndrome^7^,^8^,^9^. Epigenetic proteins can serve as potential therapeutic targets for treatment of cancer and small inhibitors targeting them are already in pre-clinical and clinical trials. dbEM provides information about 54 small molecule inhibitors of HDACs, HMTs, DNMTs and HDMs, which have been used either alone or in combination for treatment of a variety of cancers. dbEM is further linked to DrugBank and PubChem databases to provide more information about aforementioned epigenetic drugs and inhibitors.

### Integration of web tools

Many user-friendly tools have been integrated in dbEM for maximal and easy extraction of information related to epigenetic proteins in context of cancer.

### Search

We have implemented three forms of search tools namely simple search, composite search and similarity search. **Simple search** option allows user to perform search by entering a simple keyword such as protein name, location, class, subclass, domain, mutation, inhibitor, inhibitor class and their targets, which returns an aesthetic table containing all major information available about the query keyword. **Composite search** allows user to perform complex query on basis of three fields *viz*. cellular location, class and subclass by use of logical operators (AND/OR). **Similarity search** tool facilitates the user to perform similarity-based search of query protein against the epigenetic proteins included in dbEM.

### Align With Modifiers

dbEM allows user to align the query protein sequence with four different types of sequences: (i). Normal epigenetic protein sequences (ii). CCLE mutants (iii). COSMIC mutants (iv). 1000 Genome variants. Alignments can be visualized on Jalview applet and give information about consensus, quality and conservation of protein sequence. This module has an additional feature, which allows user to view dendrogram tree for query sequence with the type of sequences selected.

### Profile Based Prediction

This tool allows the user to predict whether a certain change in protein sequence would be considered as a normal variation (SNP) or cancer causing mutation. It is based on the similarity score of query sequence with HMM profiles of normal variants from 1000 Genome project and cancer mutants from CCLE and COSMIC. If the similarity score of query protein sequence is higher with HMM profile of cancer mutants than normal variants, then it will be declared as cancer causing mutation and vice-versa. This tool calculates the similarity with respective HMM profiles by use of ‘hmmsearch’ module of HMMER suite (version 3.1b1)^21^.

### Browse Section

dbEM has a powerful browsing facility, which allows the user to browse the database and acquire information using the five major modules **1. Epigenetic Modifiers:** This module provides all epigenetic proteins in a tabulated form with each of them linked to UniProt, homologous proteins, PDB and PubChem/ChEMBL bioassay links. **2. Chromosomes:** In this module epigenetic proteins have been categorized on the basis of their gene location on respective chromosomes. Circos plot depicts the chromosomal distribution, gene expression and copy number variations (CNV) of epigenetic genes (Fig. 3). **3. Frequency of Mutation:** Frequency of cancer mutations and normal variations for each protein was calculated on the basis of mutational information of epigenetic proteins from CCLE and COSMIC and variants from 1000 Genome project. Higher ratio of cancer mutation frequency to normal variant frequency for a protein marks it as a potential drug target for anticancer therapy. DNMT3A, HDAC2 and KDM6A have highest frequency of mutation in cancer as shown in Table 1. **4. Genomic Features:** This module allows user to select epigenetic proteins on the basis of certain range of genomic features related to mutation frequency, expression range and copy number variation (CNV). **5. Drugs/Inhibitors:** User can use this module to gather information about 54 molecules that are used as inhibitors or epigenetic drugs for treatment of various cancers. Information includes class, chemical class, therapeutic use, clinical trial status and mode of action of these molecules. The molecules in dbEM are linked to PubChem and DrugBank databases for detailed information.

### Information

In this section of dbEM, information about data statistics, publication, related links and acknowledgment is provided. **Data Stats:** In this module, we have incorporated the statistics of distribution of these epigenetic proteins on the basis of class, subclass, cellular location, frequency of nature of mutation and chromosomal location. **Literature:** This link provides the list of recent research articles related to epigenetics in health and disease. **Useful Links:** On this page links to various databases that have been used for data acquisitions are provided. **Acknowledgments:** In this section, authors of databases and software used in construction of dbEM are acknowledged.

### Get Data

In order to maximize the use of dbEM and to complement the scientific research community, a dedicated download page is built which allows the user to download data related to mutation, expression data, copy number variation, modeled tertiary structures/domains; sequence alignment/profiles and post-translational modifications of epigenetic proteins available in dbEM.

## Discussion

To find a cure for cancer has always been a challenging task for researchers. There is a need to study the mechanism of induction and progression of cancer in detail and to find some potential targets for drug designing. In the past decade, recent development in proteomic, transcriptomic and whole genome sequencing methodologies have lead to plethora of research articles relating epigenetic mechanisms to health and disease. Similarly, earlier studies related to epigenetic modifications, especially DNA methylation and histone modifications by activity of chromatin modifiers (such as HDACs, HMTs, HDMs, HATs, and DNMTs) in context of cancer have provided useful insights. It has been observed that any alteration to epigenetic regulation in normal tissues may lead to induction or progression of cancer by various mechanisms, for example, the activation/expression of proto-oncogenes or repression of tumor suppressor genes^27^,^28^. Small molecules that inhibit the action of certain epigenetic proteins in cancer have met success to cure cancer, for example, recently FDA approved the use of vorinostat and romidepsin (HDAC inhibitors); azacitidine and decitabine (DNMT inhibitors) for treatment of cutaneous T cell lymphoma and myelodysplastic syndrome^7^,^8^,^9^. Thus, there is a need for a database which compiles the information about various epigenetic proteins in context of cancer. Keeping this tenet in mind dbEM has been developed. It serves as a single platform where genomic data about mutation, copy number variation and expression level of epigenetic proteins from thousands of tumors and cancer cell lines (different databases and literature) have been compiled. Analysis of this data shows that some epigenetic proteins such as DNMT3A, HDAC2, KDM6A, TET2 are highly mutated in most of the cancer cell lines. Gene essentiality data about epigenetic proteins in dbEM makes it more convenient to enlist the genes whose inhibition can lead to either mitigation or eradication of cancer progression. dbEM would be highly beneficial for the researchers to analyze the genomic status of epigenetic proteins in cancer to design and discover small molecules inhibitors that could influence epigenetic regulation to cure cancer. Gene essentiality data further boosts the importance of dbEM in cancer research e.g. ACTL6A gene has high negative GARP score (-2.4) in case of pancreatic cancer cell line, HPDE. It suggests the importance of ACTL6A for the survival of HPDE cancer cell line and indicates its importance as a therapeutic target. The structural and sequence alignment information in dbEM may further aid in drug designing and screening of potential targets. In order to increase the success rate of researchers in anticancer drug designing, brief information about 54 small epigenetic drugs/ inhibitors with their targets has been included. These drugs have been used either alone or in combination for treatment of various cancers and are already in preclinical or clinical trials. Recently, NIH has received funding for screening of 1400 compounds and to assay them over thirty epigenetic enzymes, which would lead to an epigenetic drug database. In future, we would update our database and try to link the NIH epigenetic drug database with our dbEM database so as to further streamline epigenetic drug designing. dbEM also serves as a standard platform to develop similar databases for other important classes of proteins *viz*. apoptosis, protein kinases, receptor proteins and metabolic pathway proteins which may have epigenetic role in oncogenesis. Cancer specific data for these classes of proteins is also available in various repositories and can be easily integrated in a platform like dbEM. Presence of such platforms will help in deciphering the cancer mechanisms and it will open up a new arena for cancer drug development.

## Additional Information

**How to cite this article**: Singh Nanda, J. *et al*. dbEM: A database of epigenetic modifiers curated from cancerous and normal genomes. *Sci. Rep*. **6**, 19340; doi: 10.1038/srep19340 (2016).

## Figures and Tables

**Table 1:** Top 10 mutated epigenetic proteins in cancer.

S. No.	Protein	CCLE Mutation count	COSMIC Mutation count	Sum of CCLE & COSMIC count (S)	Variants Count in 1000 Genome (V)	S/V
1	DNMT3A	8	1663	1671	82	20.38
2	HDAC2	60	49	109	6	18.17
3	KDM6A	60	246	306	17	18.00
4	TET2	34	1608	1642	115	14.28
5	ARID1A	172	821	993	87	11.41
6	PRDM9	0	333	333	33	10.09
7	HDAC3	29	39	68	7	9.71
8	PRMT6	0	32	32	4	8.00
9	HDAC6	35	111	146	22	6.64
10	RBBP7	0	58	58	9	6.44
